# Docosahexaenoic acid-mediated protein aggregates may reduce proteasome activity and delay myotube degradation during muscle atrophy *in vitro*

**DOI:** 10.1038/emm.2016.133

**Published:** 2017-01-20

**Authors:** Seung Kyun Shin, Ji Hyeon Kim, Jung Hoon Lee, Young Hoon Son, Min Wook Lee, Hak Joong Kim, Sue Ah Noh, Kwang Pyo Kim, In-Gyu Kim, Min Jae Lee

**Affiliations:** 1 Department of Applied Chemistry, College of Applied Sciences, Kyung Hee University, Yongin, Korea; 2 Department of Biomedical Sciences, Seoul National University Graduate School, Seoul, Korea; 3 Department of Biochemistry and Molecular Biology, Seoul National University College of Medicine, Seoul, Korea; 4 Department of Chemistry, College of Science, Korea University, Seoul, Korea

## Abstract

Proteasomes are the primary degradation machinery for oxidatively damaged proteins that compose a class of misfolded protein substrates. Cellular levels of reactive oxygen species increase with age and this cellular propensity is particularly harmful when combined with the age-associated development of various human disorders including cancer, neurodegenerative disease and muscle atrophy. Proteasome activity is reportedly downregulated in these disease conditions. Herein, we report that docosahexaenoic acid (DHA), a major dietary omega-3 polyunsaturated fatty acid, mediates intermolecular protein cross-linkages through oxidation, and the resulting protein aggregates potently reduce proteasomal activity both *in vitro* and in cultured cells. Cellular models overexpressing aggregation-prone proteins such as tau showed significantly elevated levels of tau aggregates and total ubiquitin conjugates in the presence of DHA, thereby reflecting suppressed proteasome activity. Strong synergetic cytotoxicity was observed when the cells overexpressing tau were simultaneously treated with DHA. Antioxidant *N*-acetyl cysteine significantly desensitized the cells to DHA-induced oxidative stress. DHA significantly delayed the proteasomal degradation of muscle proteins in a cellular atrophy model. Thus, the results of our study identified DHA as a potent inducer of cellular protein aggregates that inhibit proteasome activity and potentially delay systemic muscle protein degradation in certain pathologic conditions.

## Introduction

The ubiquitin–proteasome system (UPS) is the primary cellular proteolytic machinery that regulates the homeostasis of various regulatory proteins and eliminates damaged, misfolded and mutant proteins for protein quality control.^1^ UPS substrates are covalently conjugated with ubiquitin through isopeptide bonds between the ɛ-amine in internal lysine residues and the C-terminal carboxylic group of ubiquitin. Polyubiquitinated proteins are mostly targeted to 26S proteasomes for proteolysis into small peptides of 3 to 24 amino acids.^2^ Proteasomes can also directly degrade certain substrates—mainly intrinsically unfolded or disordered proteins—without ubiquitination.^3^ Oxidatively damaged proteins compose a class of misfolded proteins in cells. Under normal conditions, intracellular oxidized proteins are rapidly recognized and degraded by cytosolic calpains,^4^ lysosomal cathepsins^5^ and the UPS in both ubiquitin-dependent and ubiquitin-independent manners to avoid their harmful consequences.^6^ The proteasome, as the primary clearance system of oxidatively damaged proteins, is responsible for the degradation of ~90% of these proteins.^5^

Reactive oxygen species (ROS) are routinely generated in cells during mitochondrial metabolic reactions and frequently produced in response to xenobiotics, cytokines, bacterial invasion and replication stress. During disease pathogenesis and the natural aging process, ROS are excessively produced and accumulated in cells. Unneutralized ROS are highly reactive in the modification of various biomolecules such as DNA, lipids and proteins. Consequently they alter signaling pathways, interfere with cellular metabolism, and cause genetic instability, epigenetic change and mutation.^7^ To prevent these harmful consequences, cells actively utilize chemical and enzymatic antioxidation mechanisms and DNA/protein repair systems.^8^ However, if oxidative damage exceeds the antioxidant capacity, ‘oxidative stress' occurs and is potentially cytotoxic, leading to cellular damage and even cell death.

Protein oxidation by ROS has garnered attention because the mechanisms through which ROS damage occurs are closely implicated in the etiology of pathological states such as age-related cancer, neurodegeneration, diabetes, cardiac diseases, excess glucocorticoids and muscle atrophy. For example, strong etiopathological links between neurodegenerative diseases and oxidative stress have been suggested, in which elevated ROS levels in the brain may trigger neurotoxic protein oxidation and the formation of nucleates for insoluble protein aggregates.^9^ These pathologic consequences may also be linked to age-related decreases in proteasome activity.^10^ Decreased rates of protein degradation and the accumulation of oxidatively damaged protein are common features in all aged cells.^11^ Several lines of evidence presented during the past two decades indicate that ROS also have key roles in both the initiation and the regulation of muscle atrophy, which results from aging, cancer, diabetes and renal failure.^12, 13^ In most types of muscle atrophy, overall rates of protein synthesis are suppressed while rates of protein degradation are elevated. The latter process is mainly mediated by upregulated UPS flux involving ubiquitin, proteasome subunits and specific E3 ubiquitin ligases of muscle proteins.^14, 15^ However, the underlying molecular mechanism connecting ROS-related signaling pathways and the activation of UPS components in atrophic muscles remains unidentified.

Docosahexaenoic acid (DHA), an omega-3 polyunsaturated fatty acid (ω3-PUFA), is a primary structural component of the human brain, skin, sperm and retina. DHA can be synthesized by mammals from other ω3-PUFA, but most of them are obtained from dietary sources, such as fish oil. Studies in cultured cells and animal models have shown evidence that ω3-PUFAs may have anti-tumor activity.^16, 17^ One hypothesis is that ω3-PUFAs in the plasma and mitochondrial membranes are highly susceptible to peroxidation by ROS, and the resulting lipid peroxides inhibit DNA synthesis and cell division.^18, 19^ Via similar chemical modifications, ω3-PUFAs can mediate the formation of protein carbonyls, which are direct markers of ROS-mediated protein oxidation and, ultimately, protein–protein cross-linked derivatives.^20^ DHA activates pro-apoptotic pathways via intracellular oxidative stress, which is more specific to cancer cells, but this effect is abolished in the presence of antioxidants.^21^

Although various beneficial effects of DHA for human health have been suggested, the current understanding of how DHA affects UPS-mediated proteolysis of oxidized proteins remains undetermined. Herein, we demonstrate that DHA increases ROS production in cells, leading to oxidative imbalance and the formation of protein aggregates both *in vitro* and in cultured cells. We also observed that DHA-mediated protein aggregates potently inhibit proteasome activity, possibly by clogging proteasome core particles, and delay the degradation of proteasome substrates. These effects lead to the development of additional cellular stress, accumulation of tau aggregates and increased sensitization to proteotoxicity. Inhibited proteasome activity by DHA resulted in significant inhibition of muscle protein degradation in myotube cells under muscle atrophic conditions. Thus, these findings reveal not only a novel molecular mechanism for DHA-induced oxidative stress, protein aggregation and proteasome activity regulation but also possible therapeutic implications for DHA in protecting cells from atrophy-induced muscle wasting.

## Materials and methods

### Fatty acids and chemical analysis of DHA

DHA (catalog number 90310, >98% purity) and arachidonic acids (90010, >98% purity) were obtained from Cayman Chemical (Ann Arbor, MI, USA). Docosanoic acids were purchased from Matreya (1035, 99% purity, State College, PA, USA). They were dissolved in absolute ethanol (Daejung, Siheung, Korea, 0005–20287) to obtain a 10 mM stock, aliquoted and stored at −20 °C until used. The DHA stock solution was diluted in culture media to generate working solutions freshly before experiment. The chemical composition and possible modification of DHA were examined upon arrival through NMR and LC/MS analysis (Supplementary Figures 1 and 2). Upon arrival or after several-months storage, no chemical modification was observed in DHA. Additional information is available in Supplementary Methods.

### Purification of the 26S human proteasome

Human 26S proteasomes were purified using a stable HEK293 cell line harboring biotin-tagged human β4 as previously described with slight modification.^6^ The cells were cultured in 15-cm culture dishes, and Dounce-homogenized in lysis buffer (100 mM NaCl, 50 mM NaH_2_PO_4_ (pH 7.5), 10% glycerol, 5 mM MgCl_2_, 0.5% NP40, 5 mM ATP, 1 mM DTT) containing protease inhibitors. After centrifugation, the supernatants were incubated with streptavidin agarose bead (Millipore, Darmstadt, Germany) for 5 h at 4 °C. The beads washed with lysis buffer and TEV buffer (50 mM Tris-HCl (pH 7.5) containing 1 mM ATP and 10% glycerol). The 26S proteasome was eluted from the beads using TEV protease (Invitrogen, Waltham, MA, USA) in TEV buffer. To cleavage proteasome from streptavidin beads, using TEV protease containing 1 mM ATP for 1 h at 30 °C and was concentrated using an Amicon ultra-spin column (Millipore). The purified proteasomes were separated by a 4–20% gradient sodium dodecyl sulfate polyacrylamide gel electrophoresis (SDS-PAGE) and routinely checked for their size and purity by the EzWay silver staining kit (Koma Biotech, Seoul, Korea) or Coomassie brilliant blue (CBB, Sigma-Aldrich, St Louis, MO, USA) staining.

### Purification of recombinant Sic1^PY^ and tau

pET21-Sic1 or pET29b-Tau was transformed in *Escherichia coli* strain BL21 (DE3) cells and purification of Sic1 or Tau was performed as previously described^6^ with some modifications. Cultures were incubated at 37 °C; when OD_600nm_ reached 0.6–0.8, 1 mM IPTG was added to each culture, and the cells were incubated overnight at room temperature. Cells were harvested in PBS containing protease inhibitor cocktails and lysed by sonication. After centrifugation and filtering the lysates, the supernatants were incubated with TALON resin (Clontech, Mountain View, CA, USA) at 4 °C for 1 h. After washing with PBS, Sic1 or Tau proteins were eluted using elution buffer (50 mM Na-phosphate (pH 7.0), 300 mM NaCl, 10% glycerol, 150 mM iminazole). The purified Sic1 or Tau protein was separated by SDS-PAGE and stained using CBB for size and purity. If necessary, sic1 or tau proteins were incubated with DHA and/or *N*-acetyl cysteine (NAC) in assay buffer (50 μM Tris-HCl (pH 7.5), 1 mM EDTA, 1 mg ml^−1^ BSA, 1 mM ATP, 1 mM DTT).

### Detection of cellular ROS

Intracellular ROS levels were monitored by treating cell permeable chloromethyl-2′,7′-dichlorodihydrofluorescein diacetate (CM-H_2_DCF-DA; Invitrogen) using fluorescence microscopic analysis. Cells attached to glass slides were incubated with 10 μM of CM-H_2_DCF-DA for 10 min in 37 °C. After washing with PBS twice, cells were fixed with 4% paraformaldehyde (pH 7.4) for 30 min, washed with PBS twice and cover-slipped in mounting medium containing 4′,6-diamidino-2-phenylindole. The images were obtained by fluorescence microscope (excitation and emission 492–495/517–527 nm). Oxidized proteins were detected using the OxyBlot protein oxidation detection kit (Millipore). Briefly, total proteins from cells were isolated after treatment with 25 μM of menadione (Sigma-Aldrich) for 2 h, and 15 μg of protein was used for derivatization with 2,4-dinitrophenyl hydrazine for 25 min. Samples were resolved by SDS-PAGE and anti-DNP antibody was used for subsequent immunoblotting.

### Western blot analysis

Whole-cell extracts were prepared as following: cells were lysed in RIPA buffer (50 mM Tris-HCl (pH 8.0), 0.5% sodium deoxycholate, 150 mM NaCl, 1% NP-40, 0.1% SDS) with 1% protease inhibitor cocktail and centrifuged to remove insoluble matter. Protein concentration of the supernatant was measured using Coomassie protein assay reagent (Pierce, Waltham, MA, USA). Supernatant fractions of an equal protein cell number, generally corresponding to 10–20 μg per lane, or 1/10 of the sample recovered from one well of a six-well plate, were separated on SDS-PAGE gels, transferred onto PVDF membrane and analyzed by conventional western blotting. The primary antibody GFP (E1T510, 1/5000) was purchased from Enogene (New York, NY, USA). Ubiquitin (sc-8017, 1/5000), glucocorticoid receptor (sc-1004, 1/1000) and myoD (sc-304, 1/1000) were purchased from Santa Cruz (Dallas, TX, USA). Tau (AHB0042, 1/5000), Alexa Fluor 568 phalloidin (A12380, 1/500) were purchased from Invitrogen. Phospho-glucocorticoid receptor (4161, 1/1000), atrogin-1 (AP2041, 1/1000) were purchased from Cell Signaling Technology (Danvers, MA, USA), ECM (Versailles, KY, USA). α-tubulin (T9026, 1/5000), β-actin (A1978, 1/10 000) and LC3 (L7543, 1/1000) were purchased from Sigma-Aldrich, respectively. αb-crystallin (ADI-SPA-223, 1/5000), α3 (BML-PW8115, 1/5000) and α7 (BML-PW8110, 1/3000) were purchased from Enzo Life Sciences (Farmingdale, NY, USA). DNP (UG9510, 1/200) was purchased from Millipore.

### Activity measurement of proteasomes using fluorogenic peptide substrates

On the basis of hydrolysis of fluorogenic succinyl-Leu-Leu-Val-Tyr-7-amido-4-methylcoumarin (suc-LLVY-AMC) peptides, Boc-Leu-Arg-Arg-AMC (Boc-LRR-AMC) and Z-Leu-Leu-Glu-AMC (Z-LLE-AMC), the chymotrypsin-like, trypsin-like and caspase-like activity of proteasomes were measured to determine their proteolytic activity. The hydrolysis assay of a fluorogenic substrate for proteasome activity was carried out using purified proteasome and 12.5 μM of a fluorogenic substrate (Enzo Life Sciences) in assay buffer (50 nM Tris-HCl (pH 7.5), 1 mM EDTA, 1 mg ml^−1^ BSA, 1 mM ATP, 1 mM DTT). Relative fluorescence unit was measured after a 30 min incubation at room temperature. To measure proteasome activity in cells, whole-cell extracts were prepared in lysis buffer (100 mM NaCl, 50 mM NaH_2_PO_4_ (pH 7.5), 10% Glycerol, 5 mM MgCl_2_, 0.5% NP40, 1 mM ATP, 1 mM DTT) containing protease inhibitors, homogenized using 10 strokes with 1 ml syringe (needle: 26G × 1/2″) and centrifuged to remove insoluble matter. The purified and whole-cell proteasome activities were monitored by measuring free AMC fluorescence on a black 96-well plate using a TECAN infinite m200 fluorometer (TECAN, Männedorf, Switzerland).

### Mammalian cell cultures and transient expression

Mammalian cells used in this study, including HEK293, HEK293-pre1-HTBH, HEK293-trex-htau40 and C2C12 cells, were grown in DMEM supplemented with 10% FBS, 2 mM glutamine, and 100 units per ml penicillin/streptomycin. Cells were maintained in a humidified incubator with 5% CO_2_ at 37 °C. For transfection, cells were treated with 1–2 μg of total plasmid DNA in a 6-well culture plate (>95% confluent or at a density of 10^6^ cells per well) for 36–48 h using Lipofectamine 3000 (Invitrogen). Cell lysates were prepared in RIPA buffer 36–48 h post transfection and were used for immunoblotting. For chase analysis, wild type and α3ΔN cells were treated with 75 μg ml^−1^ cycloheximide (Enzo Life Sciences) and samples were isolated at chase times 0, 20, 40 and 60 min after 4 h transient MG132 (AG Scientific, San Diego, CA, USA) treatment and vigorous washing with phosphate-buffered saline (PBS). For stable cell line maintenance, transfected cells were cultured with DMEM medium containing 600 mg ml^−1^ G418 (Santa Cruz) and 10% FBS. Fluorescence images were obtained after cells were extensively rinsed three times with PBS.

### Assessment of cell viability

Cell viability was measured by a modified 3-(4, 5-dimethylthiazol-2-yl)-2, 5-diphenyltetrazolium bromide (MTT) assay. Briefly, cells at ~90% confluency were treated with DHA (Cayman) or menadione at indicated various concentration for 4 h, followed by the addition of 10 μl of 5 mg ml^−1^ thiazolyl blue tetrazolium bromide (MTT, Sigma-Aldrich) solution; the mixture incubated for 2.5 h at 37 °C in a humidified atmosphere of 95% air and 5% CO_2_. After discarding the media, 200 μl of DMSO was added to solubilize the blue MTT-formazan product, and the cells were incubated for an additional 30 min at room temperature. The absorbance of the solution was read at 570 nm (test) and 630 nm (reference). Triplicate wells were assayed for each condition.

### *In vitro* ubiquitination of recombinant Sic1

Polyubiquitinated Sic1 with PY motifs (Ub-Sic1^PY^) was prepared as previously described^22^ with some modifications. Briefly, the Ub conjugation mixture contained 10 pmol Sic1^PY^, 2 pmol Uba1, 5 pmol Ubc4, 5 pmol Rsp5 and 1.2 nmol ubiquitin in a buffer of 50 mM Tris-HCl (pH 7.4), 100 mM NaCl, 1 mM DTT, 5 mM ATP and 10 mM MgCl_2_. Conjugation proceeded for 4 h at 25 °C. To purify the conjugates, they were absorbed to a Ni-NTA resin (Qiagen, Hilden, Germany), washed with buffer (50 mM Tris-HCl (pH 8.0), 50 mM NaCl, and 40% glycerol), eluted with 200 mM imidazole in wash buffer and dialyzed into wash buffer containing 10% glycerol.

### Assaying tau aggregation in cultured cells

HEK293-trex-htau40 cells were cultured as described above. Cells were treated with 500 ng ml^−1^ Dox for 24 h to induce tau expression after 48 h post transfection, lysed into buffer A (20 mM Tris, pH 7.4, 150 mM NaCl, 1% Triton X-100 and protease inhibitor cocktail), and centrifuged at 200 *g* for 15 min at 4 °C. Whole-cell lysates were washed five times with the lysis buffer and resuspended in SDS sample buffer for immunoblotting using anti-tau antibody.

### *In vitro* model of muscle atrophy

C2C12 cells were maintained to have low passage numbers and differentiated as previously described.^23^ Briefly, the medium was replaced with DMEM supplemented with 2% FBS for ~4 days. The differentiated states of the cells were monitored by myotube size and the number of nuclei per myotube. To induce muscle atrophy, various concentrations of dexamethasone (DEX, Sigma-Aldrich) for indicated times were added to the cells. Cells were either lysed with RIPA buffer to prepare whole-cell extracts or fixed with 4% paraformaldehyde for conventional immunoblotting and immunostaining analysis, respectively. Actin staining was performed by incubating the fixed cells with 1:40 diluted phalloidin-Alexa Fluor 568 dye in PBST for 20 min.

### Statistical analysis

Statistical significance of difference between various groups was determined by one-way analysis of variance (ANOVA) followed by the Bonferroni *post hoc* test in most data. Differences were considered to be significant *P*<0.05. The Michaelis–Menten kinetic parameters were obtained by fitting the experiment data to a nonlinear regression model, using GraphPad Prism 5 (GraphPad Inc, La Jolla, CA, USA)

## Results

### DHA increases intracellular ROS levels and decreases cellular UPS flux

**Figure 1:**
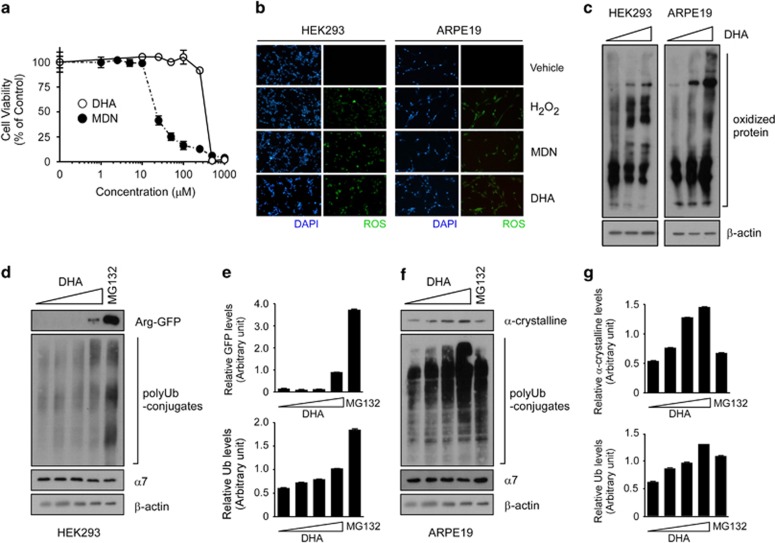
Docosahexaenoic acid (DHA) produces reactive oxygen species (ROS) and perturbs ubiquitin-proteasome system (UPS) flux in cells. (**a**) The cytotoxicity of ROS inducers such as DHA and menadione on HEK293 cells was measured with MTT assay. Values represent means±s.d. (*n*=4). (**b**) Cellular ROS were detected with fluorescence microscopy using CM-H_2_DCF-DA. HEK293 and ARPE19 cells were treated with DHA (200 μM), H_2_O_2_ (200 μM) or menadione (MDN, 10 μM) for 4 h before treatment with 10 μM H_2_ DCF-DA for 10 min and counterstaining with 4′,6-diamidino-2-phenylindole (DAPI). (**c**) After HEK293 and ARPE19 cells were incubated with DHA (200 μM) for 4 h, whole-cell extracts (WCEs) were prepared and treated with 2,4-dinitrophenyl hydrazine (DNPH). Immunoblotting analysis was performed with anti-DNP antibody to visualize the total oxidized proteins. β-actin, loading control. (**d**) HEK293-derived cells stably expressing Arg-GFP were treated with DHA (0, 50, 100 or 250 μM) or MG132 (10 μM) for 4 h. WCEs were analyzed with SDS–PAGE/immunoblotting (IB) using the indicated antibodies. Accumulated polyubiquitin (polyUb) conjugates indicate downregulated ubiquitin–proteasome system (UPS) flux in the cells. (**e**) Quantification of Arg-GFP or total polyUb conjugate levels in **d**. (**f**) As in **d**, except that α-crystalline was transiently overexpressed in ARPE19 cells. (**g**) Quantification of α-crystalline or total polyUb conjugate levels in **f**.

To examine the effects of DHA on cellular oxidative stress and its consequences, we initially examined HEK293 cell viability after treatment with DHA and menadione, another ROS inducer. Although DHA is reportedly cytotoxic in various cancer cells, we observed only the mild effects of DHA on HEK293 viability; whereas, menadione showed significant cytotoxicity at ~25 μM, DHA had no noticeable cytotoxicity at concentrations up to ~250 μM (Figure 1a). However, when we treated working concentrations of menadione (10 μM) and DHA (200 μM) to the cells for 4 h, a cell-permeable ROS probe, CM-H_2_DCF-DA, showed significant amounts of intracellular ROS (Figure 1b). The extent of ROS induction by DHA was comparable to that by menadione or H_2_O_2_ (200 μM). Therefore, DHA appeared to be a potent ROS inducer with less cytotoxicity. Moreover, we observed that DHA also significantly increased ROS levels in ARPE19 retina pigment epithelial cells (Figure 1b). The cell death induced by high concentrations of DHA appeared to be mediated via caspase 7 or PARP-dependent apoptosis, but not by caspase 3 (Supplementary Figure 3).^24^ These cells have a hyperactive UPS and an efficient defense system against cellular oxidative stress,^25^ and therefore may function as a superior model to assess the effect of DHA on oxidative stress.

Next, we examined the effects of ROS on the generation of oxidized proteins in both types of cells. These proteins compose an important subset of the misfolded substrates of proteasomes that accumulate under various pathological conditions. After ROS were induced by DHA, total oxidized proteins were labeled with 2,4-dinitrophenyl hydrazine and visualized by recognizing their modified carbonyl groups through anti-DNP immunoblotting. Both HEK293 and ARPE19 cells showed strikingly elevated levels of oxidized proteins that rose in a largely dose-dependent manner (Figure 1c). To the contrary, saturated fatty acid docosanoic acids (C22:0) did not elevate protein aggregates levels *in vitro* or cellular ubiquitin-conjugates levels (Supplementary Figure 4), which indicates that perturbation of the cellular UPS system by DHA may be specific and direct effects of DHA, instead of secondary consequences through oxidative stress-mediated signaling pathways. Together, these results suggest that DHA effectively induces oxidative stress and leads to the generation of ROS and oxidized proteins in cells.

To investigate whether DHA-mediated increases in oxidized proteins affect cellular UPS flux, we initially examined the steady states of arginine-green fluorescent protein (Arg-GFP), an artificial model substrate of the N-end rule pathway, a subset of UPS-dependent proteolysis.^26^ As previously observed,^27, 28^ Arg-GFP has a short half-life (<5 min) in HEK293 cells under normal conditions and is virtually undetectable with immunoblotting (Figures 1d and e). These characteristics were completely reversed after treatment with MG132 (10 μM, 4 h), a proteasome inhibitor. MG132 treatment also resulted in, as expected, rapid accumulation of total polyubiquitin conjugates in the cells. We observed significant increases in both Arg-GFP and total polyubiquitin conjugate levels after cells were treated with 200 μM DHA for 4 h (Figures 1d and e). These increases were not due to the changes in proteasome biogenesis in the cells, as determined by measuring levels of proteasome subunit α7 (Figure 1d). Therefore, these results indicate that oxidative stress induced by DHA may inhibit proteasome activity and elevate levels of unstable Arg-GFP proteins and other endogenous proteasome substrates.

The effects of DHA were then investigated by using α-crystalline protein and ARPE19 cells. Crystalline proteins are soluble and compose ~90% of the mature lens. The aggregation of these lens proteins into high-molecular-weight complexes accounts for the loss of transparency to visible light and decreased visual acuity in patients with cataracts.^29^ Although MG132 had only a mild stabilizing effect, treatment with DHA significantly blocked the degradation of α-crystalline in a dose-dependent manner (Figures 1f and g). Similarly, DHA elevated levels of total polyubiquitinated proteins in ARPE19 cells more markedly than MG132, which indicated that overall UPS flux was severely impaired, possibly because ARPE19 retina epithelial cells have more adaptive defense mechanisms to cellular oxidative stress.^30^ Together, our data indicate that DHA induces intracellular ROS generation and oxidative stress, which inhibit UPS substrate degradation in cells.

### DHA inhibits proteasome function via the formation of protein aggregates

**Figure 2:**
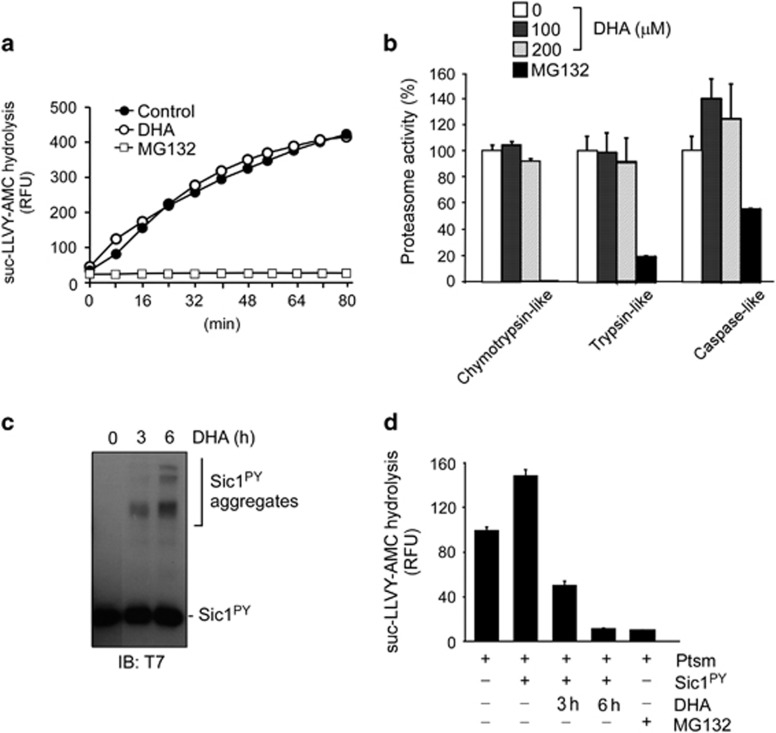
Docosahexaenoic acid (DHA)-mediated protein aggregates, not DHA itself, inhibit proteasome activity. (**a**) Proteasome activity was kinetically monitored through hydrolysis of the fluorogenic peptides suc-LLVY-AMC (12.5 μM) in the presence of DHA (100 μM) or MG132 (10 μM). (**b**) The three different proteolytic sites in the purified human proteasome were measured by using the fluorogenic substrates suc-LLVY-AMC (for chymotrypsin-like activity), Boc-LRR-AMC (for trypsin-like) and Z-LLE-AMC (for caspase-like). DHA (0, 100 or 200 μM) or MG132 (10 μM) was added in the hydrolysis reactions, and relative proteasome activity was quantified after the reactions were quenched after 30 min. (**c**) *In vitro* Sic1^PY^ (100 nM) aggregation mediated by DHA (200 μM for 3 or 6 h). T7-tagged Sic1^PY^ proteins were analyzed by using SDS-PAGE/IB with T7 antibodies. (**d**) The effect of DHA-mediated aggregation of Sic1^PY^ on proteasome activity was examined with suc-LLVY-AMC (12.5 μM) hydrolysis. Recombinant Sic1^PY^ proteins (100 nM) were pre-incubated with 200 μM of DHA at 30 °C for 6 h and added to the hydrolysis reactions. MG132 (10 μM) was used, which indicated the baseline activity of purified proteasomes. RFU, relative fluorescence unit. Ptsm, 26S human proteasomes. Values represent means±s.d. (*n*=3).

To further determine the effect of DHA on proteasomes and its underlying mechanism, we used succinyl-Leu-Leu-Val-Tyr-7-amido-4-methylcoumarin (suc-LLVY-AMC), a highly sensitive fluorogenic substrate of 26S proteasomes, and human proteasomes affinity-purified from an HEK293-derived cell line stably expressing biotin-tagged β4 subunits.^31, 32^ The fluorescence intensity gradually increased over time as a result of suc-LLVY-AMC hydrolysis, which indicated the normal degradation activity of purified proteasomes (Figure 2a). Direct addition of 100 μM DHA to the reaction had negligible effects on proteasome activity, whereas treatment with MG132 completely blocked their proteolytic activity (Figure 2a). Proteasomes contain six proteolytic active sites (two trypsin-like, two chymotrypsin-like, and two caspase-like, in specificity) in the interior of their β-subunit rings.^33^ Currently, the most widely used proteasome inhibitors, including MG132, are most effective in blocking chymotrypsin-like activity, but their efficacies are highly variable and concentration dependent at other sites.^34^ We observed that, similar to chymotrypsin-like β5 activity, for which suc-LLVY-AMC hydrolysis is specific, trypsin-like β2 and caspase-like β1 activities were virtually unaffected by DHA, as measured by *N*-tert-Boc-Leu-Arg-Arg-7-amido-4-methylcoumarin (Boc-LRR-AMC) and benzyloxycarbonyl-Leu-Leu-Glu-methylcoumarylamide (Z-LLE-AMC) hydrolysis, respectively (Figure 2b). These results indicate that DHA has no effect on proteasome activity and that the altered UPS flux observed in the cultured cells is not the direct effect of DHA itself but likely a secondary consequence of oxidative stress, which turned out to be the protein aggregates formed by DHA-mediated oxidative stress.

We next attempted to examine the effect of oxidized proteins on proteasome activity by using a proteasome substrate, polyubiquitinated Sic1^PY^ (Ub-Sic1^PY^). Sic1 is a CDK inhibitor from *Saccharomyces cerevisiae*, and its modified form, Sic1^PY^, has been used in *in vitro* ubiquitination and degradation assays because the PY element functions as a signal for polyubiquitination with mixed ubiquitin linkage types leading to proteolysis.^22, 35^ We found that excess amounts of DHA resulted in the formation of significant amounts of Sic1^PY^ aggregates after 3 h of incubation *in vitro* (Figure 2c). DHA completely blocked the ubiquitination of Sic1^PY^, where the reaction was mediated by UBA1 (as an E1 enzyme), UBC4 (E2) and RSP5 (E3) in test tubes (Supplementary Figure 5). Then we observed that DHA-mediated Sic1^PY^ aggregates drastically reduced the hydrolysis activity of human proteasomes, which was unaffected by DHA alone (Figure 2d). The inhibitory efficiency was proportional to the degree of Sic1^PY^ aggregation. After 6 h of incubation with DHA and Sic1^PY^, the resulting 100 nM aggregated proteins blocked proteasome activity as potently as 100-fold more of MG132 (10 μM). These data provide strong evidence that DHA as an oxidative stress inducer inhibits proteasome activity indirectly through the formation of protein aggregates that may compensate for the normal degradatory function of proteasomes.

### DHA-induced oxidative stress and subsequent proteasome inhibition are relieved by an antioxidant *in vitro* and in cultured cells

**Figure 3:**
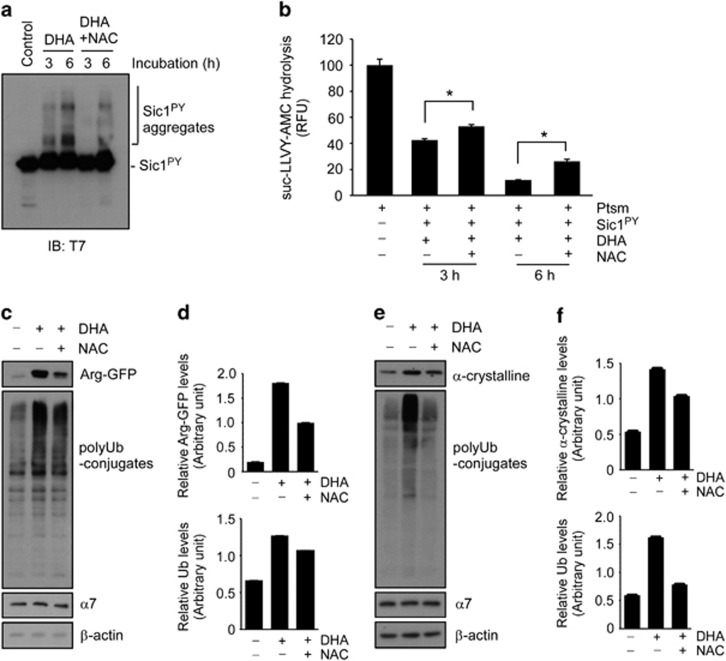
An antioxidant reverses the inhibitory effects of docosahexaenoic acid (DHA) on proteasome activity by reducing protein aggregate formation. (**a**) DHA (200 μM) was co-incubated with recombinant Sic1^PY^ (100 nM) in the presence or absence of *N*-acetyl cysteine (NAC, 200 μM) at 30 °C for the indicated time periods. Sic1^PY^ aggregation was analyzed with SDS-PAGE/IB against T7 antibodies. (**b**) Proteasome activity was measured with suc-LLVY-AMC hydrolysis (for 30 min) in the presence of Sic1^PY^ (100 nM), which was reacted with DHA (200 μM), NAC (200 μM), or a combination thereof for 3 or 6 h. Values represent means±s.d. *P<0.05 (*n*=3, ANOVA with Bonferroni's multiple comparison test). (**c**) HEK293 cells stably expressing Arg-GFP were treated with DHA (200 μM) and NAC (200 μM) for 4 h, and whole-cell extracts (WCEs) were prepared and analyzed with SDS-PAGE/IB using the antibodies indicated. (**d**) Quantification of **c**. (**e**) ARPE19 cells were transiently transfected with α-crystalline for 24 h and analyzed with SDS-PAGE/IB. (**f**) Quantification of α-crystalline or total polyUb conjugate levels in **e**.

To further determine that excess DHA potently induces protein oxidation and aggregation, which leads to the inhibition of proteasome function, we examined whether treatment with an antioxidant reverses DHA-mediated proteasome dysfunction. We began by using the Sic1^PY^ aggregation reaction with DHA as shown in Figure 2c and found that the oligomeric assembly of Sic1^PY^ was significantly reduced when the same concentration of antioxidant NAC(200 μM) was added to the *in vitro* reaction (Figure 3a). Treatment with NAC also rescued DHA-mediated proteasome inhibition as measured by suc-LLVY-AMC hydrolysis (Figure 3b). These results strongly indicate that the protein aggregation and subsequent proteasome activity dysfunction induced by DHA are mediated mainly by protein oxidation.

To explore the possibility of applying antioxidants from test tubes to cultured cells, we initially examined the effects of NAC in HEK293 cells stably expressing Arg-GFP. As in Figure 1d, otherwise short-lived Arg-GFP in the cells became stabilized in the presence of DHA with increased levels of polyubiquitin conjugates (Figures 3c and d). On the contrary, the application of NAC to the cells significantly reduced Arg-GFP levels with lower levels of polyubiquitin conjugates. The rescue effect of NAC against DHA-induced oxidative stress was also observed in ARPE19 cells transiently overexpressing α-crystalline (Figures 3e and f). Therefore, our results indicate that the antioxidant not only suppressed protein aggregation and proteasome inhibition by DHA *in vitro* but also effectively delayed these processes in cultured cells expressing proteasome substrates.

### Effects of DHA on tau aggregation and proteasome inhibition

**Figure 4:**
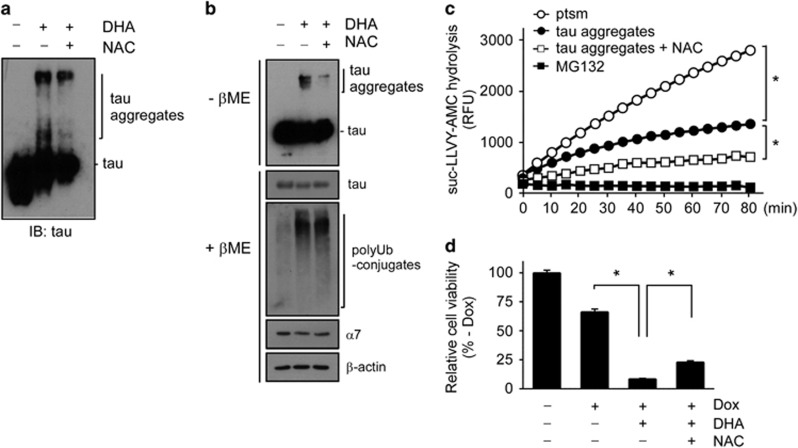
Effects of docosahexaenoic acid (DHA) on tau aggregation and proteotoxic stress-mediated cytotoxicity. (**a**) Recombinant htau40 proteins (100 nM) were incubated with DHA (200 μM), *N*-acetylcysteine (NAC; 200 μM), or both. Tau aggregation was analyzed with SDS-PAGE/IB using tau antibodies under reducing conditions. (**b**) Monomeric and aggregated forms of tau proteins were observed in whole-cell extracts (WCEs) of inducible tau cells after doxycycline (Dox) treatment (500 ng ml^−1^) for 24 h. Cells were treated with DHA (200 μM), NAC (200 μM), or both for 4 h before sample collection. Protein samples were prepared under non-reducing conditions (without β-mercaptoethanol (-βME)) or reducing conditions (with β-mercaptoethanol (+βME)), and analyzed with SDS-PAGE/IB. (**c**) Proteasome activity was monitored with suc-LLVY-AMC hydrolysis after incubating htau40 (100 nM) with DHA (200 μM) in the presence or absence of NAC (200 μM). Values represent means±s.d. (*n*=3). *P<0.05 (*n*=3, two-way ANOVA). (**d**) DHA significantly sensitized the proteotoxicity of the inducible tau cell line, which was induced by excess amounts of overexpressed htau40. The inducible tau cell line was treated with 500 ng ml^−1^ Dox for 24 h in the presence or absence of DHA (50 μM) and NAC (50 μM). Cell viability was measured with the MTT assay. Values represent means±s.d. (*n*=3). *P<0.01 (one-way ANOVA with Bonferroni's multiple comparison test).

The oxidation of proteins under oxidative stress has been suggested to play a critical role in various human diseases including Alzheimer's disease (AD). A key pathological hallmark of AD is the formation of cytosolic inclusions with tau proteins in neurons.^36^ Tau proteins are extremely soluble under normal conditions and do not spontaneously aggregate. Although the precise mechanism of the conversion from soluble tau monomers to insoluble aggregates remains to be elucidated, oxidative modifications may induce the cross-linking of covalent bonds in tau, thereby leading to fibril formation and insolubility. Because we observed DHA involvement in protein oxidation and aggregation, we assessed whether DHA induces tau aggregation *in vitro*. DHA markedly facilitated the *in vitro* aggregation of recombinant htau40, the longest isoform of human tau, and this process was effectively reversed by treatment with NAC (Figure 4a). Tau proteins are substrates of 26S proteasomes, and impaired proteasomal activity may be related to the progression of AD.^37^ Therefore, DHA-mediated tau aggregation may be reciprocally involved in the progression of AD via modulated proteasome activity.

We then used a HEK293-derived cell line that expresses htau40, the longest isoform of human tau, upon doxycycline (Dox) induction (an inducible tau cell line).^38^ These cells produced significant amounts of SDS-resistant tau aggregates with DHA in culture (Figure 4b). Unlike in the *in vitro* experiments, high-molecular-weight tau species appeared only under the non-reducing conditions, which suggests that the tau aggregates in the cells originate from the intermolecular disulfide bonds of htau40. The initial disulfide bonds between tau proteins may be mediated by DHA-induced cellular ROS to form a nucleating structure that leads to the formation of higher-order multimers and aggregates via intermolecular self-assembly.^39^ When NAC was added to the cells with DHA, the levels of tau aggregates in the inducible tau cells significantly decreased (Figure 4b). Similar to the results obtained by using Sic1^PY^ aggregates, human proteasomes with DHA-induced tau aggregates showed significantly reduced proteasome activity (Figure 4c). However, NAC could partially rescue this activity. Consistent with the previous findings,^40^ cells treated with DHA showed increased levels of LC3-II and p62 in a dose-dependent manner (Supplementary Figure 6). Bafilomycin A_1_ further elevated these autophagic marker levels, suggesting a positive role of DHA on autophagic flux. The consequence of DHA-induced autophagy and its crosstalk with the ubiquitin–proteasome system in the cells remains to be determined.

Because the aggregation of recombinant tau proteins is cytotoxic, we anticipated that treatment with DHA would aggravate proteotoxic stress in the cells, possibly via proteasome inhibition. In the inducible tau cell line, excess amounts of htau40 proteins induced by 500 ng ml^−1^ Dox for 24 h resulted in significant cell death (~34% Figure 4d). We further observed that the HEK293-derived tauopathy model cells showed synergetic cytotoxicity with DHA: ~91% of the tau expressing-cells died after treatment with 100 μM DHA, which is completely nontoxic under normal conditions (Figure 1a). Moreover, cells co-treated with NAC showed significantly alleviated cytotoxicity induced by tau and DHA. Therefore, DHA appears to induce significant synergic cytotoxicity when cells are under proteotoxic stress, and antioxidant *N*-acetyl cysteine can alleviate this harmful consequence of DHA-mediated proteasome inhibition.

### Beneficial effects of DHA on muscle atrophy via delayed proteasomal degradation of muscle proteins

**Figure 5:**
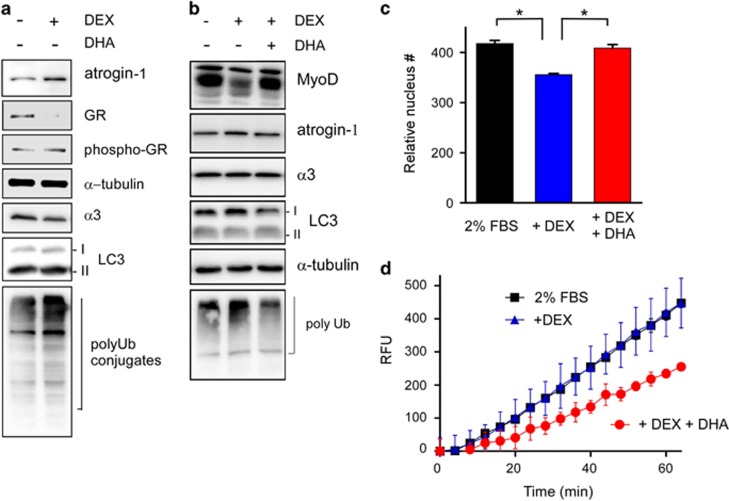
Anti-atrophic effects of docosahexaenoic acid (DHA) on C2C12-based muscle atrophy model cells. (**a**) Differentiated C2C12 cells were treated with dexamethasone (DEX; 10 μM, 24 h) to induce muscle atrophy. Whole-cell extracts (WCEs) were then prepared and analyzed with SDS-PAGE/IB. (**b**) As in **a**, except that cells were co-treated with 25 μM DHA. (**c**) Nucleus numbers in the C2C12 cells under different conditions (10 μM DEX and 50 μM DHA for 24 h) were quantitated and compared. Values represent means±s.d. (*n*=3). *P<0.01 (one-way ANOVA with Bonferroni's multiple comparison test). (**d**) suc-LLVY-AMC hydrolysis assay using WCEs in the presence and absence of DHA (100 μM). DHA-treated C2C12 cells showed significantly lower proteasome activity.

Because a loss of skeletal muscle loss or muscle atrophy usually results from elevated proteolysis by the UPS,^41, 42^ we hypothesized that suppressed proteasome activity by DHA may benefit cells by delaying muscle protein degradation. To examine the anti-atrophic effect of DHA on skeletal muscle cells, we first differentiated C2C12 myoblasts to myotubes with a low concentration of serum in the media and treated them with DEX to induce muscle atrophy.^23^ We found that treatment of differentiated C2C12 cells with DEX (10 μM, 24 h) led to significantly decreased levels of glucocorticoid receptor and MyoD but increased levels of phosphorylated glucocorticoid receptor and atrogin-1, a known atrophy-associated E3 Ub ligase (Figure 5a). These results confirmed that our system was a highly reliable *in vitro* muscle atrophy model. The differentiated myotubes were readily distinguishable with phalloidin staining of assembled F-actin and 4′,6-diamidino-2-phenylindole counterstaining owing to their characteristic morphology and multinucleated characteristics (data not shown).

When DEX was added to the C2C12 cells, collapsed myotubes were accompanied by reduced levels of MyoD (Figure 5b). In the presence of DHA, however, atrophic C2C12 cells were recovered to have the basal levels of MyoD, atrogin-1 and polyubiquitinated proteins as before DEX was added (Figure 5b). No cytotoxicity from DHA was observed in the atrophic model of C2C12 cells (data not shown). Moreover, the DEX-induced fragmentation of myotubes was also significantly delayed when DHA was cotreated to the differentiated C2C12 cells (Figure 5c). This is accompanied by significantly reduced proteasome activity, which is measured by using whole-cell extracts, in C2C12 cells (Figure 5d), indicating that the post-translational turnover of muscle proteins was impaired by DHA-induced proteasome inhibition. Therefore, changes in muscle protein degradation appeared to be the consequences of reduced proteasome activity. Together, these results suggest that DHA effectively attenuates proteasome-mediated muscle protein degradation under atrophic conditions.

## Discussion

**Figure 6:**
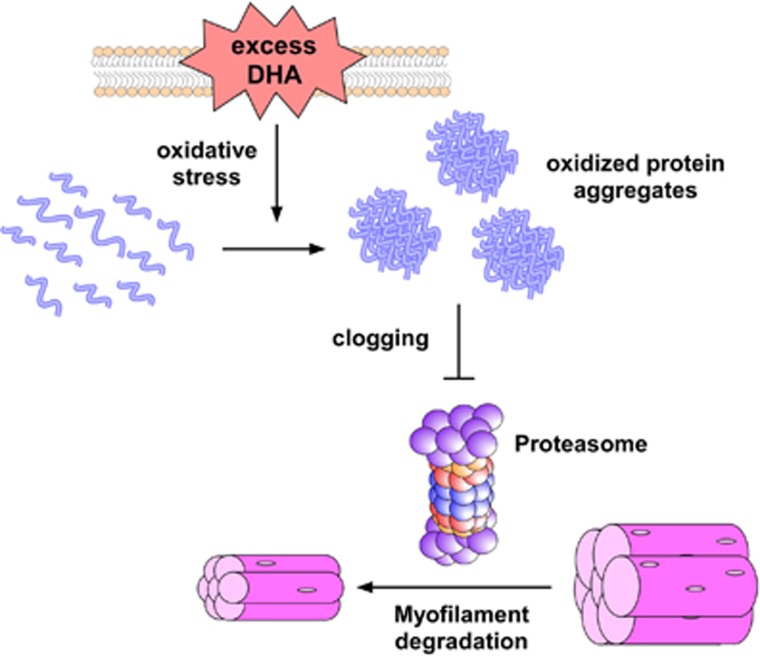
Illustrated model of the effects of docosahexaenoic acid (DHA) on proteasome activity and muscle atrophy. Under physiological conditions, 26S proteasomes in the cell tightly regulate the levels of oxidatively damaged proteins to prevent their harmful effects on cell viability. Elevated concentrations of DHA may result in extensive oxidative stress likely through lipid peroxidation and oxidize various cellular proteins in a promiscuous manner. The consequent oxidized proteins may function as excess loads to proteasomes and inhibit their degradatory activity by clogging the catalytic chambers. Therefore, in cells under proteotoxic stress with tau aggregates, DHA may aggravate cellular toxicity by elevating oxidative stress and inhibiting proteasome activity. Under muscle atrophy and other pathologically conditions associated with increased cellular ubiquitin–proteasome system flux, DHA may be beneficial to cells by delaying proteasomal degradation of substrate proteins.

Herein, we reported that the ω-3 PUFA DHA induces cellular oxidative stress more effectively than previously envisioned. Subsequently, treatment with DHA may also inhibit proteasome activity in cells via clogging by an excess of resultant oxidized and aggregated proteins. Proteasome dysfunction may then generate additional oxidative stress and even more protein aggregates in cells. Because proteasomes are a major degradation machinery for oxidatively damaged and aggregation-prone proteins including tau, DHA appears to exert synergic toxic effects particularly in cells under stress conditions. As illustrated in Figure 6, the inhibitory effects of DHA on proteasomes may be beneficial in muscle cells under atrophic stress because they can delay proteasomal degradation of muscle proteins. ω3-PUFAs have long been known to attenuate the development of muscle atrophy—not only in murine models but also in cachectic cancer patients.^43, 44, 45^ Although their effects have largely been attributed to downregulated cellular UPS flux, the underlying molecular mechanism has remained unclear. Our results demonstrate that the beneficial effects of DHA in muscle atrophy may be due to its capability to produce adequate oxidative stress, which can attenuate cellular proteasome activity without affecting cell viability.

ω3-PUFA such as DHA has been known for decades to enhance the efficacy of anti-cancer reagents and to decrease the severity of secondary cancer complications such as cachexia.^46, 47^ However, the exact molecular mechanism of these beneficial effects of DHA is still elusive. Given the biochemical mechanism of DHA-mediated anti-atrophic effects via proteasomes suggested in this study, therapeutic strategies targeting upstream E3 ubiquitin ligases, such as atrogin-1 and MuRF1, will likely have only limited capacity. Oxidative stress has been implicated in aging and other human diseases such as neurodegeneration. Therefore, cells appear to have diverse and sometimes contradictory response mechanisms toward oxidative stress depending on the type, stress levels and possibly age itself. Therefore, the positive and negative biochemical effects of this dietary compound on human health should be investigated further. The potential of DHA as a anti-atrophic agent should be explored more through global analysis of cellular oxidative stress and subsequent cellular ubiquitome changes.

## References

[bib1] Finley D, Ciechanover A, Varshavsky A. Ubiquitin as a central cellular regulator. Cell 2004; 116: S29–S32. 15055578 10.1016/s0092-8674(03)00971-1

[bib2] Pickart CM, Cohen RE. Proteasomes and their kin: proteases in the machine age. Nat Rev Mol Cell Biol 2004; 5: 177–187. 14990998 10.1038/nrm1336

[bib3] Baugh JM, Viktorova EG, Pilipenko EV. Proteasomes can degrade a significant proportion of cellular proteins independent of ubiquitination. J Mol Biol 2009; 386: 814–827. 19162040 10.1016/j.jmb.2008.12.081 PMC2649715

[bib4] Pariat M, Carillo S, Molinari M, Salvat C, Debussche L, Bracco L et al. Proteolysis by calpains: a possible contribution to degradation of p53. Mol Cell Biol 1997; 17: 2806–2815. 9111352 10.1128/mcb.17.5.2806 PMC232132

[bib5] Taggart CC, Lowe GJ, Greene CM, Mulgrew AT, O'Neill SJ, Levine RL et al. Cathepsin B, L, and S cleave and inactivate secretory leucoprotease inhibitor. J Biol Chem 2001; 276: 33345–33352. 11435427 10.1074/jbc.M103220200

[bib6] Lee BH, Lee MJ, Park S, Oh DC, Elsasser S, Chen PC et al. Enhancement of proteasome activity by a small-molecule inhibitor of USP14. Nature 2010; 467: 179–184. 20829789 10.1038/nature09299 PMC2939003

[bib7] Sies H. Oxidative stress: from basic research to clinical application. Am J Med 1991; 91: 31S–38S. 10.1016/0002-9343(91)90281-2 1928209

[bib8] Sies H. Oxidative stress: oxidants and antioxidants. Exp Physiol 1997; 82: 291–295. 9129943 10.1113/expphysiol.1997.sp004024

[bib9] Zhu X, Raina AK, Lee HG, Casadesus G, Smith MA, Perry G. Oxidative stress signalling in Alzheimer's disease. Brain Res 2004; 1000: 32–39. 15053949 10.1016/j.brainres.2004.01.012

[bib10] Goldberg AL. Protein degradation and protection against misfolded or damaged proteins. Nature 2003; 426: 895–899. 14685250 10.1038/nature02263

[bib11] Cuervo AM, Bergamini E, Brunk UT, Droge W, Ffrench M, Terman A. Autophagy and aging: the importance of maintaining ‘clean' cells. Autophagy 2005; 1: 131–140. 16874025 10.4161/auto.1.3.2017

[bib12] Powers SK, Kavazis AN, DeRuisseau KC. Mechanisms of disuse muscle atrophy: role of oxidative stress. Am J Physiol Regul Integr Comp Physiol 2005; 288: R337–R344. 15637170 10.1152/ajpregu.00469.2004

[bib13] Lecker SH, Jagoe RT, Gilbert A, Gomes M, Baracos V, Bailey J et al. Multiple types of skeletal muscle atrophy involve a common program of changes in gene expression. FASEB J 2004; 18: 39–51. 14718385 10.1096/fj.03-0610com

[bib14] Solomon V, Baracos V, Sarraf P, Goldberg AL. Rates of ubiquitin conjugation increase when muscles atrophy, largely through activation of the N-end rule pathway. Proc Natl Acad Sci USA 1998; 95: 12602–12607. 9770532 10.1073/pnas.95.21.12602 PMC22877

[bib15] Jagoe RT, Goldberg AL. What do we really know about the ubiquitin-proteasome pathway in muscle atrophy? Curr Opin Clin Nutr Metab Care 2001; 4: 183–190. 11517350 10.1097/00075197-200105000-00003

[bib16] Sala-Vila A, Calder PC. Update on the relationship of fish intake with prostate, breast, and colorectal cancers. Crit Rev Food Sci Nutr 2011; 51: 855–871. 21888535 10.1080/10408398.2010.483527

[bib17] MacLean CH, Newberry SJ, Mojica WA, Khanna P, Issa AM, Suttorp MJ et al. Effects of omega-3 fatty acids on cancer risk: a systematic review. JAMA 2006; 295: 403–415. 16434631 10.1001/jama.295.4.403

[bib18] Cornwell DG, Huttner JJ, Milo GE, Panganamala RV, Sharma HM, Geer JC. Polyunsaturated fatty acids, vitamin E, and the proliferation of aortic smooth muscle cells. Lipids 1979; 14: 194–207. 423721 10.1007/BF02533871

[bib19] Huttner JJ, Gwebu ET, Panganamala RV, Milo GE, Cornwell DC, Sharma HM et al. Fatty acids and their prostaglandin derivatives: inhibitors of proliferation in aortic smooth muscle cells. Science 1977; 197: 289–291. 877555 10.1126/science.877555

[bib20] Stadtman ER. Protein oxidation and aging. Free Radic Res 2006; 40: 1250–1258. 17090414 10.1080/10715760600918142

[bib21] Ding WQ, Lind SE. Phospholipid hydroperoxide glutathione peroxidase plays a role in protecting cancer cells from docosahexaenoic acid-induced cytotoxicity. Mol Cancer Ther 2007; 6: 1467–1474. 17431126 10.1158/1535-7163.MCT-06-0608

[bib22] Choi WH, de Poot SAH, Lee JH, Kim JH, Han DH, Kim YK et al. Open-gate mutants of the mammalian proteasome show enhanced ubiquitin-conjugate degradation. Nat Commun 2016; 7: 10963. 26957043 10.1038/ncomms10963 PMC4786872

[bib23] Son YH, Lee SJ, Lee KB, Lee JH, Jeong EM, Chung SG et al. Dexamethasone downregulates caveolin-1 causing muscle atrophy via inhibited insulin signaling. J Endocrinol 2015; 225: 27–37. 25688118 10.1530/JOE-14-0490

[bib24] Wojcik C, Lohe K, Kuang C, Xiao Y, Jouni Z, Poels E. Modulation of adipocyte differentiation by omega-3 polyunsaturated fatty acids involves the ubiquitin-proteasome system. J Cell Mol Med 2014; 18: 590–599. 24834523 10.1111/jcmm.12194 PMC4000111

[bib25] Zhang X, Zhou J, Fernandes AF, Sparrow JR, Pereira P, Taylor A et al. The proteasome: a target of oxidative damage in cultured human retina pigment epithelial cells. Invest Ophthalmol Vis Sci 2008; 49: 3622–3630. 18408178 10.1167/iovs.07-1559 PMC2694183

[bib26] Lee JH, Jiang Y, Kwon YT, Lee MJ. Pharmacological Modulation of the N-End Rule Pathway and its Therapeutic Implications. Trends Pharmacol Sci 2015; 36: 782–797. 26434644 10.1016/j.tips.2015.07.004 PMC4641009

[bib27] Jiang Y, Choi WH, Lee JH, Han DH, Kim JH, Chung YS et al. A neurostimulant para-chloroamphetamine inhibits the arginylation branch of the N-end rule pathway. Sci Rep 2014; 4: 6344. 25212999 10.1038/srep06344 PMC4161967

[bib28] Jiang Y, Pore SK, Lee JH, Sriram S, Mai BK, Han DH et al. Characterization of mammalian N-degrons and development of heterovalent inhibitors of the N-end rule pathway. Chem Sci 2013; 4: 3339–3346.

[bib29] Moreau KL, King JA. Protein misfolding and aggregation in cataract disease and prospects for prevention. Trends Mol Med 2012; 18: 273–282. 22520268 10.1016/j.molmed.2012.03.005 PMC3621977

[bib30] McGreal RS, Kantorow WL, Chauss DC, Wei J, Brennan LA, Kantorow M. alphaB-crystallin/sHSP protects cytochrome c and mitochondrial function against oxidative stress in lens and retinal cells. Biochim Biophys Acta 2012; 1820: 921–930. 22521365 10.1016/j.bbagen.2012.04.004 PMC3362689

[bib31] Hough R, Pratt G, Rechsteiner M. Purification of two high molecular weight proteases from rabbit reticulocyte lysate. J Biol Chem 1987; 262: 8303–8313. 3298229

[bib32] Lee JH, Shin SK, Jiang Y, Choi WH, Hong C, Kim DE et al. Facilitated Tau Degradation by USP14 Aptamers via Enhanced Proteasome Activity. Sci Rep 2015; 5: 10757. 26041011 10.1038/srep10757 PMC4455164

[bib33] Finley D. Recognition and processing of ubiquitin-protein conjugates by the proteasome. Annu Rev Biochem 2009; 78: 477–513. 19489727 10.1146/annurev.biochem.78.081507.101607 PMC3431160

[bib34] Kisselev AF, Callard A, Goldberg AL. Importance of the different proteolytic sites of the proteasome and the efficacy of inhibitors varies with the protein substrate. J Biol Chem 2006; 281: 8582–8590. 16455650 10.1074/jbc.M509043200

[bib35] Han DH, Na HK, Choi WH, Lee JH, Kim YK, Won C et al. Direct cellular delivery of human proteasomes to delay tau aggregation. Nat Commun 2014; 5: 5633. 25476420 10.1038/ncomms6633

[bib36] Mandelkow EM, Mandelkow E. Tau in Alzheimer's disease. Trends Cell Biol 1998; 8: 425–427. 9854307 10.1016/s0962-8924(98)01368-3

[bib37] Lee MJ, Lee JH, Rubinsztein DC. Tau degradation: the ubiquitin-proteasome system versus the autophagy-lysosome system. Prog Neurobiol 2013; 105: 49–59. 23528736 10.1016/j.pneurobio.2013.03.001

[bib38] Bandyopadhyay B, Li G, Yin H, Kuret J. Tau aggregation and toxicity in a cell culture model of tauopathy. J Biol Chem 2007; 282: 16454–16464. 17428800 10.1074/jbc.M700192200

[bib39] Sahara N, Maeda S, Murayama M, Suzuki T, Dohmae N, Yen SH et al. Assembly of two distinct dimers and higher-order oligomers from full-length tau. Eur J Neurosci 2007; 25: 3020–3029. 17561815 10.1111/j.1460-9568.2007.05555.x

[bib40] Jing K, Song KS, Shin S, Kim N, Jeong S, Oh HR et al. Docosahexaenoic acid induces autophagy through p53/AMPK/mTOR signaling and promotes apoptosis in human cancer cells harboring wild-type p53. Autophagy 2011; 7: 1348–1358. 21811093 10.4161/auto.7.11.16658 PMC3242799

[bib41] Tisdale MJ. The ubiquitin-proteasome pathway as a therapeutic target for muscle wasting. J Support Oncol 2005; 3: 209–217. 15915823

[bib42] Tisdale MJ. Mechanisms of cancer cachexia. Physiol Rev 2009; 89: 381–410. 19342610 10.1152/physrev.00016.2008

[bib43] Wigmore SJ, Barber MD, Ross JA, Tisdale MJ, Fearon KC. Effect of oral eicosapentaenoic acid on weight loss in patients with pancreatic cancer. Nutr Cancer 2000; 36: 177–184. 10890028 10.1207/S15327914NC3602_6

[bib44] Burns CP, Halabi S, Clamon GH, Hars V, Wagner BA, Hohl RJ et al. Phase I clinical study of fish oil fatty acid capsules for patients with cancer cachexia: cancer and leukemia group B study 9473. Clin Cancer Res 1999; 5: 3942–3947. 10632323

[bib45] Wigmore SJ, Ross JA, Falconer JS, Plester CE, Tisdale MJ, Carter DC et al. The effect of polyunsaturated fatty acids on the progress of cachexia in patients with pancreatic cancer. Nutrition 1996; 12: S27–S30. 8850216 10.1016/0899-9007(96)90014-3

[bib46] Tisdale MJ. Inhibition of lipolysis and muscle protein degradation by EPA in cancer cachexia. Nutrition 1996; 12: S31–S33. 8850217 10.1016/0899-9007(96)90015-5

[bib47] Vaughan VC, Hassing MR, Lewandowski PA. Marine polyunsaturated fatty acids and cancer therapy. Br J Cancer 2013; 108: 486–492. 23299528 10.1038/bjc.2012.586 PMC3593545

