# Supplementary figures and images for: Docosahexaenoic acid-mediated protein aggregates may reduce proteasome activity and delay myotube degradation during muscle atrophy in vitro
Source: Exp Mol Med. 2017 Jan 20;49(1):e287–. doi: 10.1038/emm.2016.133 (PMC5291838; doi:10.1038/emm.2016.133)

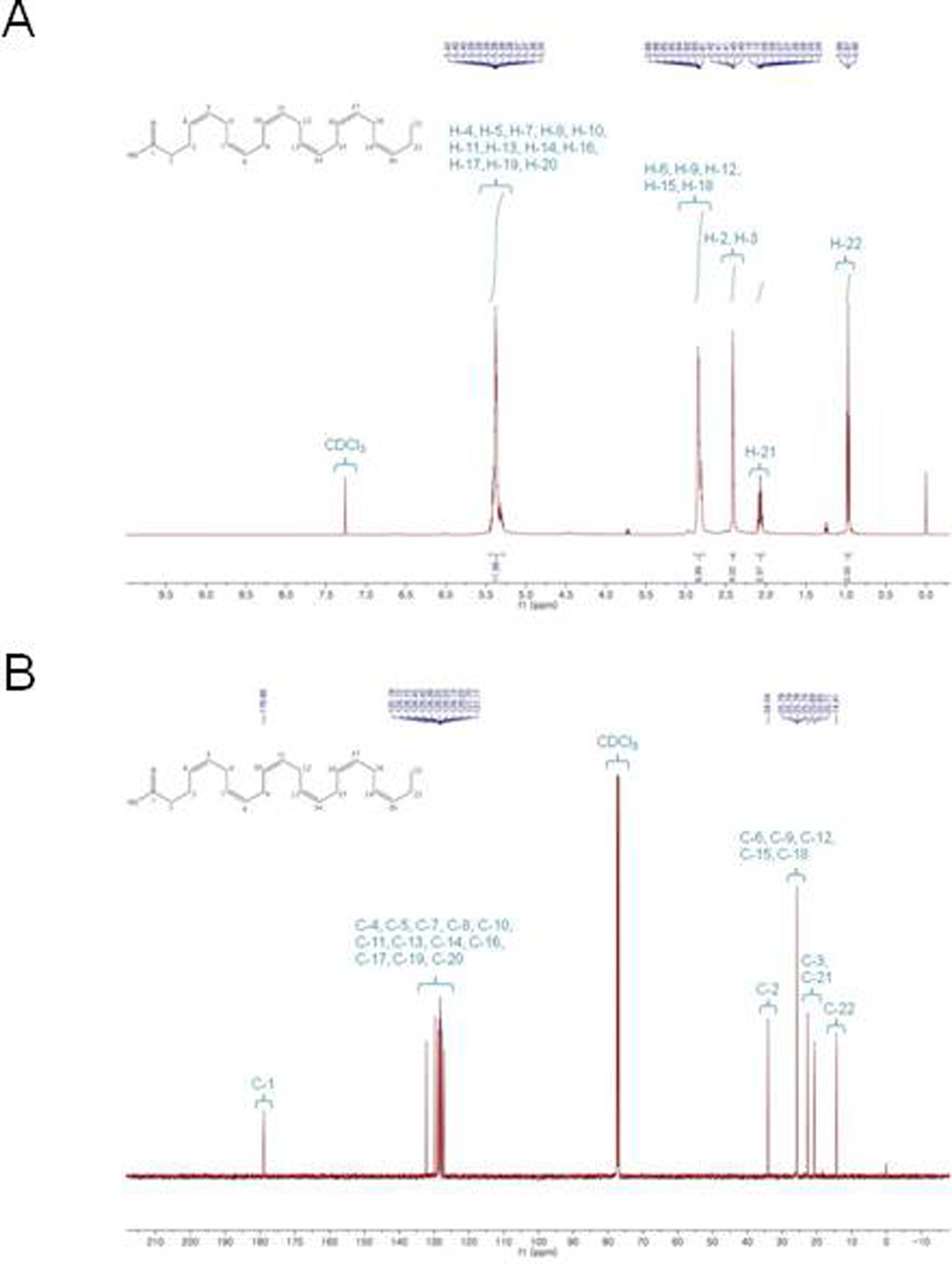

Supplement: Supplementary Figure 1 [file emm2016133x1.tif]

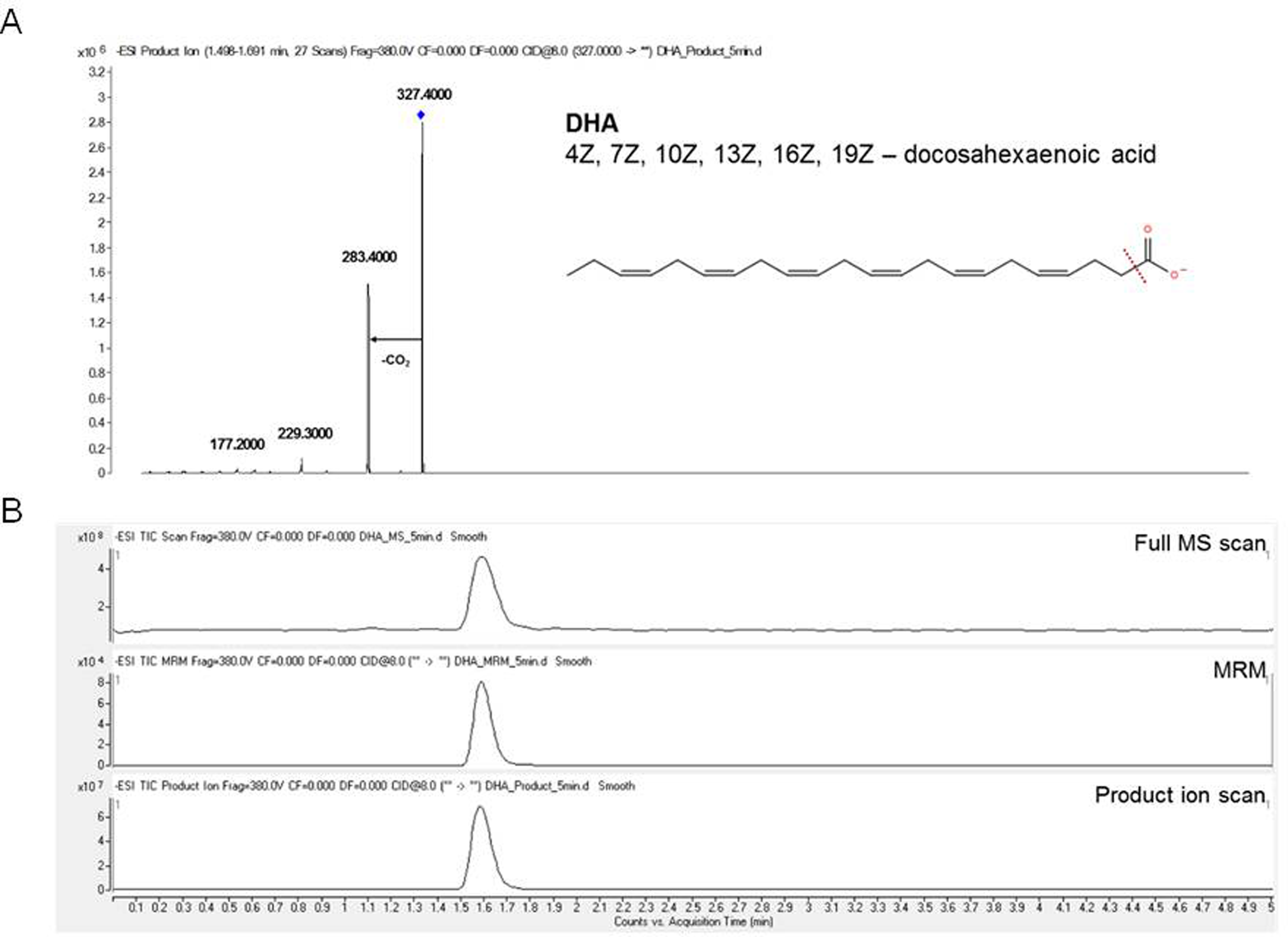

Supplement: Supplementary Figure 2 [file emm2016133x2.tif]

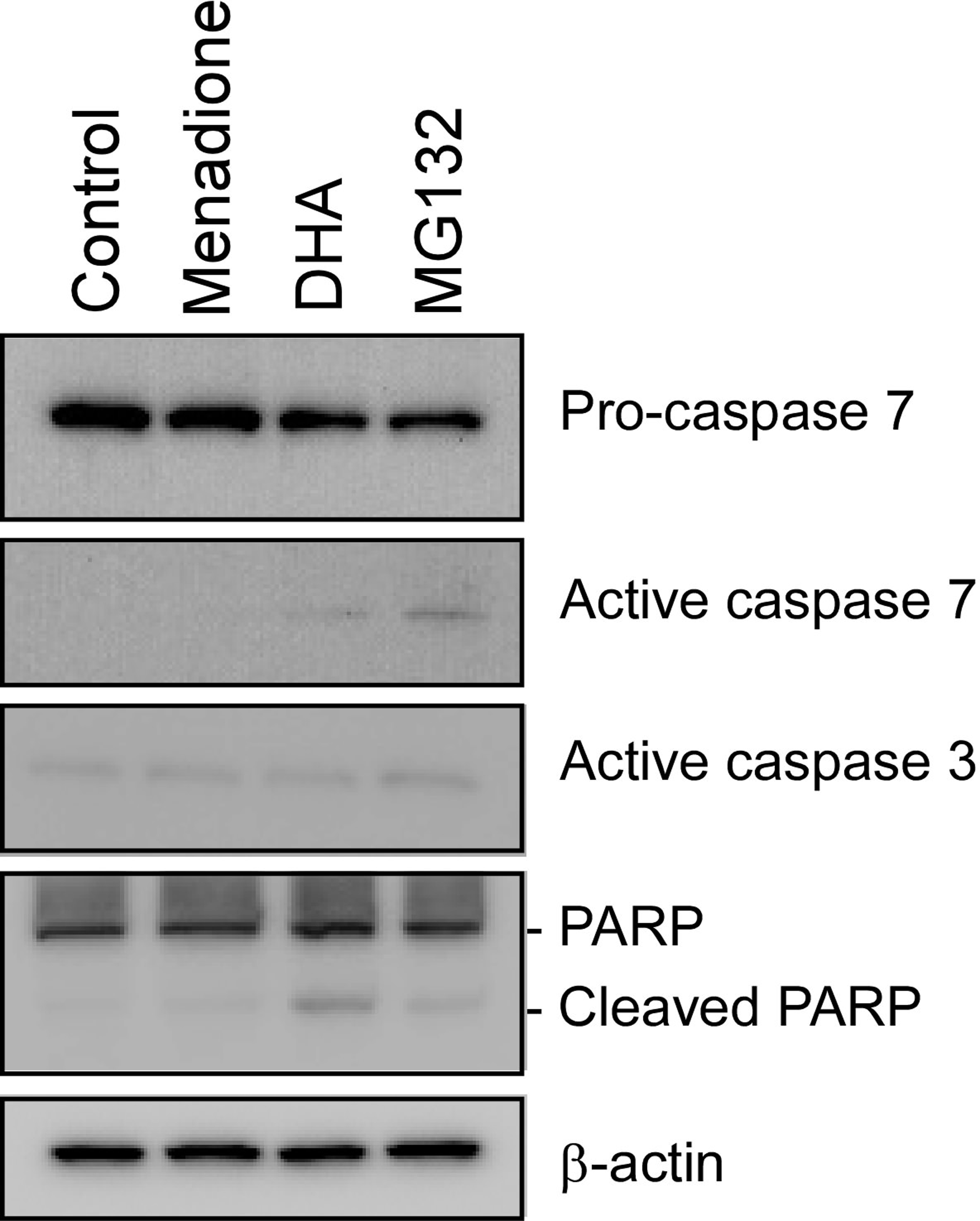

Supplement: Supplementary Figure 3 [file emm2016133x3.tif]

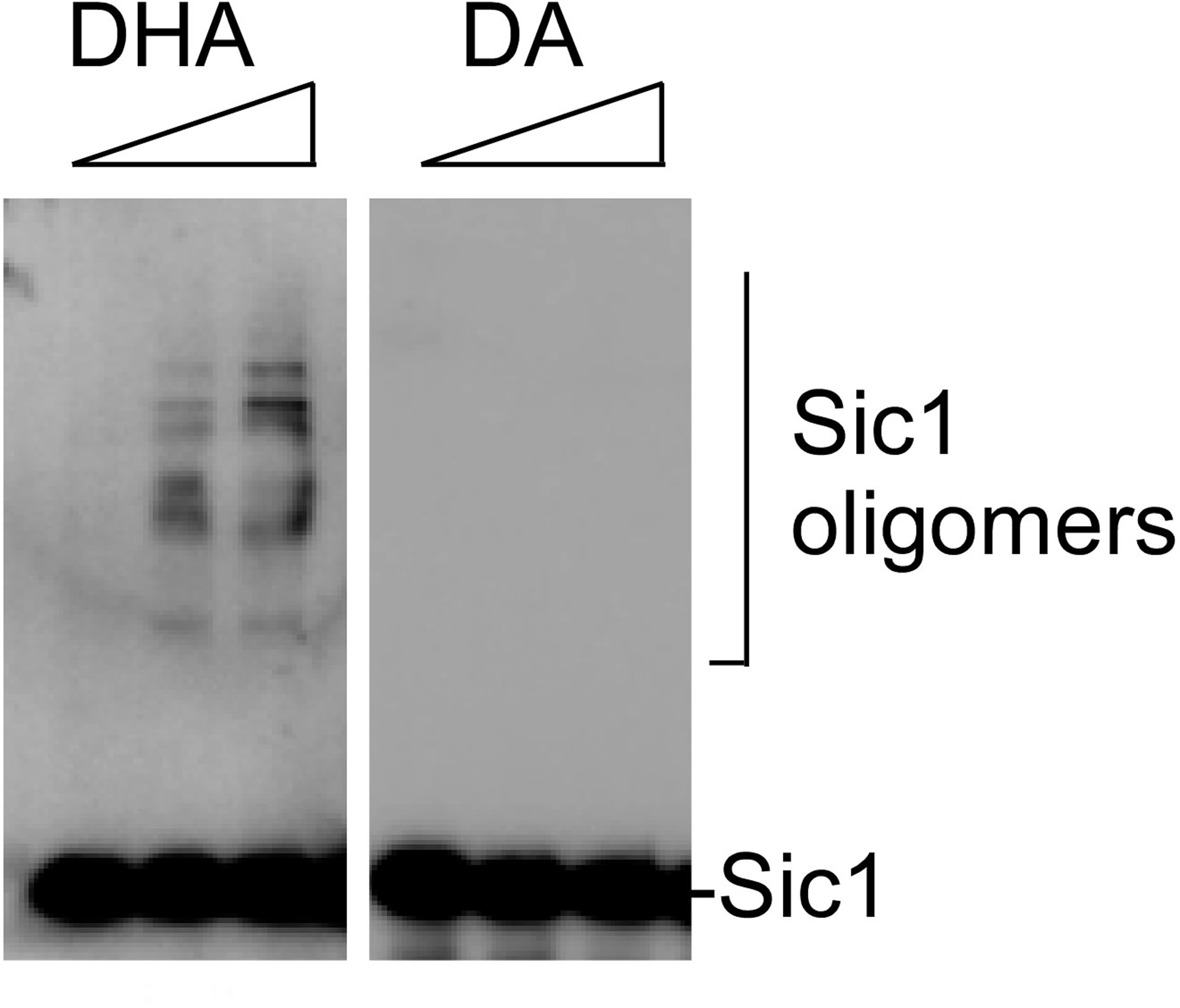

Supplement: Supplementary Figure 4 [file emm2016133x4.tif]

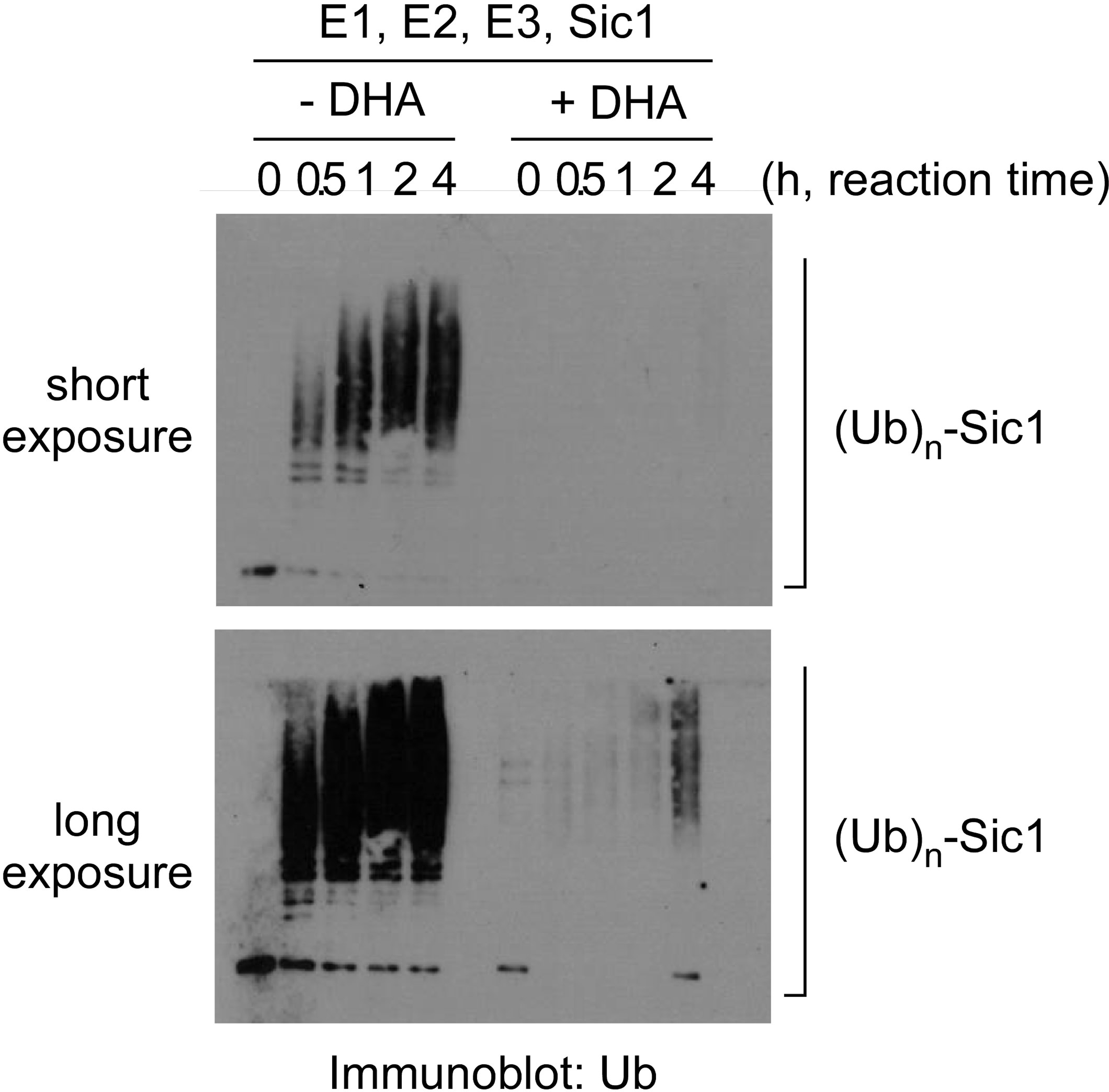

Supplement: Supplementary Figure 5 [file emm2016133x5.tif]

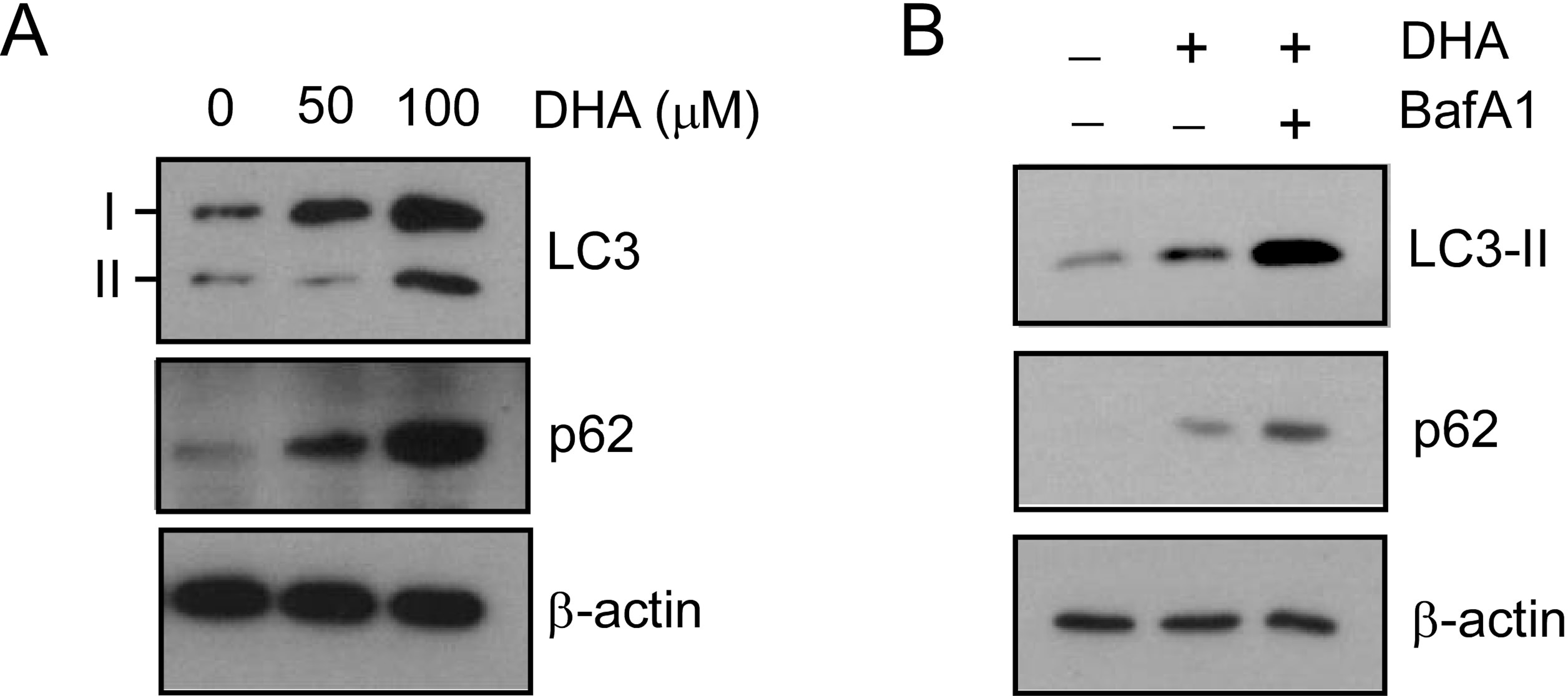

Supplement: Supplementary Figure 6 [file emm2016133x6.tif]
