# Supplementary material for: Docosahexaenoic acid-mediated protein aggregates may reduce proteasome activity and delay myotube degradation during muscle atrophy in vitro
Source: Exp Mol Med. 2017 Jan 20;49(1):e287–. doi: 10.1038/emm.2016.133 (PMC5291838; doi:10.1038/emm.2016.133)
Supplement: Supplementary Information [file emm2016133x7.docx]

*Supplementary Legends and Methods*

**Docosahexaenoic Acid-Mediated Protein Aggregates May Reduce Proteasome Activity and Delay Myotube Degradation during Muscle Atrophy *In Vitro***

Seung Kyun Shin, Ji Hyeon Kim, Jung Hoon Lee, Young Hoon Son, Min Wook Lee, Hak Joong Kim, Sue Ah Noh, Kwang Pyo Kim,In-Gyu Kim, and Min Jae Lee

**Supplementary Figure Legends**

**Supplementary Figure 1**. ^1^H-NMR (A) and ^13^C-NMR (B) spectroscopic data of DHA. The sample was highly pure, in which no significant oxidation or other chemical modification was observed in DHA. Additional information is available in Supplementary Methods.

**Supplementary Figure 2**. LC/MS analysis of DHA. (A) Consistent with the standard spectrum of DHA obtained from the LIPID MAPS (www.lipidmaps.org), the major peak at m/z = 327 was observed along with the minor peak at m/z = 283, which was generated by the carboxyl group fragmentation during MS, in the product ion scan results. (B) No contamination or modification of DHA was observed in three scan modes (full MS scan mode, MRM mode, and product ion scan mode). Negative ion MS mode was used and the retention time of DHA was 1.6 min. Additional information is available in Supplementary Methods. TIC, total ion count. MRM, multiple reaction monitoring.

**Supplementary Figure 3**. DHA increased caspase 7 and PARP activity. HEK293 cells were treated with non-lethal doses of menadione (10 μM), DHA (200 μM) and MG132 for 4 h (10 μM), and the activities of caspase-3, -7, and PARP were determined by immunoblot analysis. While menadione had little effects on apoptosis, DHA and MG132 significantly upregulated the levels of active caspase 7 and PARP, which suggest the DHA-induced apoptosis in HEK293 cells.

**Supplementary Figure 4**. Docosanoic acid (DA, C22:0), the saturated fatty acid control of DHA, did not induce Sic1 protein aggregation *in vitro*. *In vitro* Sic1^PY^ (100 nM, T7-tagged) aggregation mediated by DHA (200 μM) or DA (200 μM) for 0, 3 or 6 h reactions was monitored by using SDS-PAGE/IB with T7 antibodies.

**Supplementary Figure 5**. *In vitro* ubiquitination of Sic1 was performed with UBA1, UBC4, RSP5, and Ub in the presence and absence of DHA. Polyubiquitinated Sic (Ub_n_-Sic1) was collected after indicated times and analyzed by Ub immunoblotting.

**Supplementary Figure 6**. The inhibition of proteasome by DHA is accompanied with the changed autophagic flux. (A) Accumulated LC3-II and p62 in HEK293 cells after treatment with 0, 50, 100 μM DHA for 4 h. (B) Bafilomycin A_1_ (BafA1, 100 nM for 4 h) further potentiated the increased LC3-II and p62 levels by DHA (100 μM), suggesting a positive role of DHA on autophagic flux.

**Supplementary Methods**

**NMR analysis of DHA**

For evaluation of the purity of DHA used in this study, the compound was dissolved in CDCl_3_, and ^1^H- and ^13^C-NMR spectra were collected using a Bruker AVANCE III HD instrument in the NMR laboratory of Center for Molecular Spectroscopy and Dynamics, Institute for Basic Science, in Korea University.

^1^H-NMR (500 MHz, CDCl_3_) δ 5.35–5.42 (m, 12H), 2.81–2.86 (m, 10H), 2.40–2.42 (m, 4H), 2.07 (pd, *J* = 7.5, 1.4 Hz, 2H), 0.97 (t, *J* = 7.5 Hz, 3H).

^13^C-NMR (125 MHz, CDCl_3_) δ 178.86, 132.19, 129.72, 128.72, 128.45, 128.42, 128.39, 128.25, 128.23, 128.13, 128.03, 127.70, 127.17, 34.04, 25.790, 25.787, 25.779, 25.74, 25.69, 22.65, 20.71, 14.41.

**LC-MS analysis of DHA**

The LC analysis of DHA was conducted with Agilent 1290 infinity series LC instrument (Agilent Technologies, USA) combined with binary pump (G4220A), an autosampler (G4226A), a thermostat (G1330B) and a column compartment (G1316C). The autosampler and column oven were set at temperature of 40 °C and 4 °C, respectively. Hypersil GOLD column (2.1 X 100 mm ID; 1.9 mm Thermo scientific) was used for the separation of DHA. The mobile phase solvent A consisted of 0.1% (v/v) formic acid and 20 mmol/L ammonium formate in acetonitrile/methanol/water mixture (19 : 19 : 2) and the mobile phase solvent B consisted of 0.1 % (v/v) formic acid and 20 mmol/L ammonium formate in isopropanol. The solvent A and B was mixed in the ratio of 95:5 and the mixture was used for isocratic flow for 5 min. The flow rate of the mobile phase was 0.25 mL per min. The MS analysis was carried out on a triple quadrupole mass spectrometer (QQQ LC-MS 6490 series, Agilent Technologies) combined with an ESI source which provides high sensitivity by iFunnel technology consisting of three components such as a hexabore capillary, Agilent Jet Stream technology, and a dual ion funnel. The condition of typical operating source for MS scan in the negative ion ESI mode were optimized as follows: capillary voltage 4000 V, nozzle voltage 500 V. The nebulizer was set at 40 psi and the nitrogen drying gas was set at a flow rate of 13 L/min and the temperature was maintained at 250 °C. Three scan modes were used for determining purity of DHA. In the full MS scan mode, all substance in the sample were analyzed without fragmentation. In product ion scan mode, the specific precursor ion with m/z 327 was fragmented in collision cell for structure analysis. MRM was performed to validate the compound identity. For collision-induced dissociation (CID) experiments, the precursor ion was selected in the quadrupole analyzer and the product ions were analyzed using another quadrupole analyzer. Collision gas was ultra-pure nitrogen. The collision energy of 8 eV optimized for intact free fatty acid was applied for DHA.
